# Characterization and macrophages immunomodulatory activity of two water-soluble polysaccharides from *Abrus cantoniensis*

**DOI:** 10.3389/fnut.2022.969512

**Published:** 2022-08-22

**Authors:** Dongshuai Qu, Shuaitao Lian, Hongjie Hu, Wenjing Sun, Hongbin Si

**Affiliations:** ^1^State Key Laboratory for Conservation and Utilization of Subtropical Agro-Bioresources, College of Animal Science and Technology, Guangxi University, Nanning, China; ^2^DanAg Agritech Consulting Co. Ltd., Zhengzhou, China; ^3^Guangxi Key Laboratory of Agricultural Resources Chemistry and Biotechnology, College of Biology & Pharmacy, Yulin Normal University, Yulin, China

**Keywords:** *Abrus cantoniensis* polysaccharide, purification, physicochemical properties, immunoregulation, structure

## Abstract

The study aims to elucidate the physicochemical properties and immunomodulatory activity of two polysaccharides (ACP_*t*0_ and ACP_*t*2_) from *Abrus cantoniensis*. Results revealed that ACP_*t*0_ with a molecular weight of 26.0 kDa, was mainly composed of glucose (83.1%) and galactose (6.1%), and that ACP_*t*2_ with a molecular weight of 145.6/8.9 kDa, consisted of galactose (25.6%), galacturonic acid (22.2%), arabinos (16.6%) and galactose (11.0%) respectively. AFM and Congo red experiments suggested that ACP_*t*0_ and ACP_*t*2_ might be spherical particles with triple-helix conformation in aqueous solution. ACP_*t*0_ and ACP_*t*2_ exhibited immunomodulatory activity by promoting the proliferation, augmenting pinocytic and phagocytic capacities, releasing immunoactive molecules such as ROS, NO, iNOS, TNF-α, IL-6 and IL-1β, upregulation of the mRNA levels of corresponding cytokines in macrophages. Moreover, ACP_*t*0_ and ACP_*t*2_ were recognized by toll-like receptor 4 (TLR4) and exerted immunomodulatory effects via activating Myeloid differentiation factor 88 (MyD88), mitogen-activated protein kinases (MAPKs) and serine/threonine kinase (Akt) signaling pathways in macrophages. Notably, ACP_*t*2_ had higher immunomodulatory activity than ACP_*t*0_. Based on the present findings, ACP_*t*0_ and ACP_*t*2_ could be explored as an active component of immunomodulators in the food and pharmaceutical fields.

## Introduction

In recent decades, extensive studies have found that natural active polysaccharides isolated from various plants, especially herbal medicine, possessed rich biological activities, such as immunoregulation, anti-tumor, anti-oxidation, anti-virus, hypolipidemic, hypoglycemic and bacteriostatic activities (1–7). With the continuous research on function, increasing attention has been focused on the immunomodulatory activities of plant polysaccharides, which have become a research hotspot (8, 9).

Both innate and adaptive immunity constitute the immune system of mammals, which is closely related to body health (10). Macrophages play crucial roles in innate immunity, and can exert immune function by engulfing and destroying pathogenic substances directly, including tumor cells or infectious microbes (11). Meanwhile, macrophages can also aid in fighting against infection and inflammation indirectly by releasing bioactive molecules such as nitric oxide (NO), reactive oxygen species (ROS), tumor necrosis factor (TNF)-α, interleukin (IL)-1β, IL-6, IL-8, and IL-10 (12, 13). Therefore, the activation of macrophages is a promising approach for enhancing host immune abilities. In addition, it is reported that macrophages were activated by recognizing and binding to specific receptors. Toll-like receptor (TLR) is a key pattern recognition receptor, and some polysaccharides exert biological effects by acting on TLR2 or TLR4. *Isaria cicadae Miquel* polysaccharide can recognize TLR4 and trigger several intracellular signaling pathways (14). The effect of CPE-II on RAW264.7 is related to TLR4 and TLR2 (15). Thus, macrophages are usually used as ideal cell models to evaluate the immunomodulatory properties of polysaccharides (16).

*Abrus cantoniensis*, traditional Chinese herbal medicine, belongs to the Abrus genus in the Leguminosae family, and is widely cultivated in Guangdong, Guangxi and Hunan in Southern China. *Abrus cantoniensis* has been widely used as a folk medicine for centuries and is an important ingredient in the “Jigucao capsules.” In Guangdong areas, people use this medicinal and edible plant to make herbal teas, beverages and healthy food, such as the red-canned tea “Wong Lo Kat.” Total flavonoid extracts from *Abrus cantoniensis*, have potential anti-inflammatory effects against xylene-induced ear swelling and cotton ball granuloma in mice (17). *Abrus cantoniensis* flavonoid could improve ethanol-induced gastric ulcers and CCl_4_-induced hepatitis in mice (18, 19). The saponins of *Abrus cantoniensis* exerted potent inhibitory effects on hepatitis B virus replication *in vivo* and *in vitro* (20).

Until now, studies on *Abrus cantoniensis* polysaccharides (ACP) have mainly focused on extraction and isolation, and information on the immunomodulatory activities is not available, especially their structural features. Therefore, the purification, characterization and immunomodulatory activity of ACP *in vitro* were studied. In this study, two fractions named ACP_*t*0_ and ACP_*t*2_ were isolated and purified from *Abrus cantoniensis*. Their physicochemical properties include molecular weight, monosaccharide composition, absorption characteristic of infrared, spatial structure and AFM. Moreover, their immunomodulatory activities were investigated *in vitro* by using the macrophage model. This study will enhance our understanding of the characterization and bioactivities of ACP, which can be applied as a potential bioactive ingredient for immunomodulators in the food and pharmaceutical fields.

## Materials and methods

### Materials and reagents

*Abrus cantoniensis* were provided by the Guangxi Dahong Pharmaceutical Co., Ltd. (Hechi, Guangxi, China). After being crushed and sun-dried, the material was stored at 4°C.

Dimethyl sulfoxide (DMSO), DEAE cellulose-52, lipopolysaccharide (LPS), ROS assay Kit, RPMI 1640 medium and neutral red were acquired from Solarbio Biotechnology Co., Ltd. (Beijing, China). Monosaccharide standards (glucose, mannose, arabinose, galactose, fucose, rhamnose, glucuronic acid, galacturonic acid, glucosamine, Galactosamine, xylose), trifluoroacetic acid (TFA), 1-phenyl-3-methyl-5-pyrazolone (PMP) were purchased from Sigma (St. Louis, MO, United States). The Vybrant phagocytosis assay kit (V-6694) was obtained from Thermo Fisher Scientific (Waltham, United States). The ELISA kits for mouse tumor necrosis factor (TNF-α), interferon (IFN-γ), interleukin (IL-6, IL-1β) were provided by Jiangsu Jingmei Biotechnology Co., Ltd (Jiangsu, China). The NO and Inducible nitric oxide synthase (iNOS) detecting kit were acquired from Nanjing Jiancheng Institute of Biotechnology (Nanjing, China). Trizol reagent, RealStar Green Fast Mixture and First-strand cDNA Synthesis Mix were bought from GenStar Co., Ltd. (Beijing, China).

### Extraction and purification of polysaccharides

**FIGURE 1:**
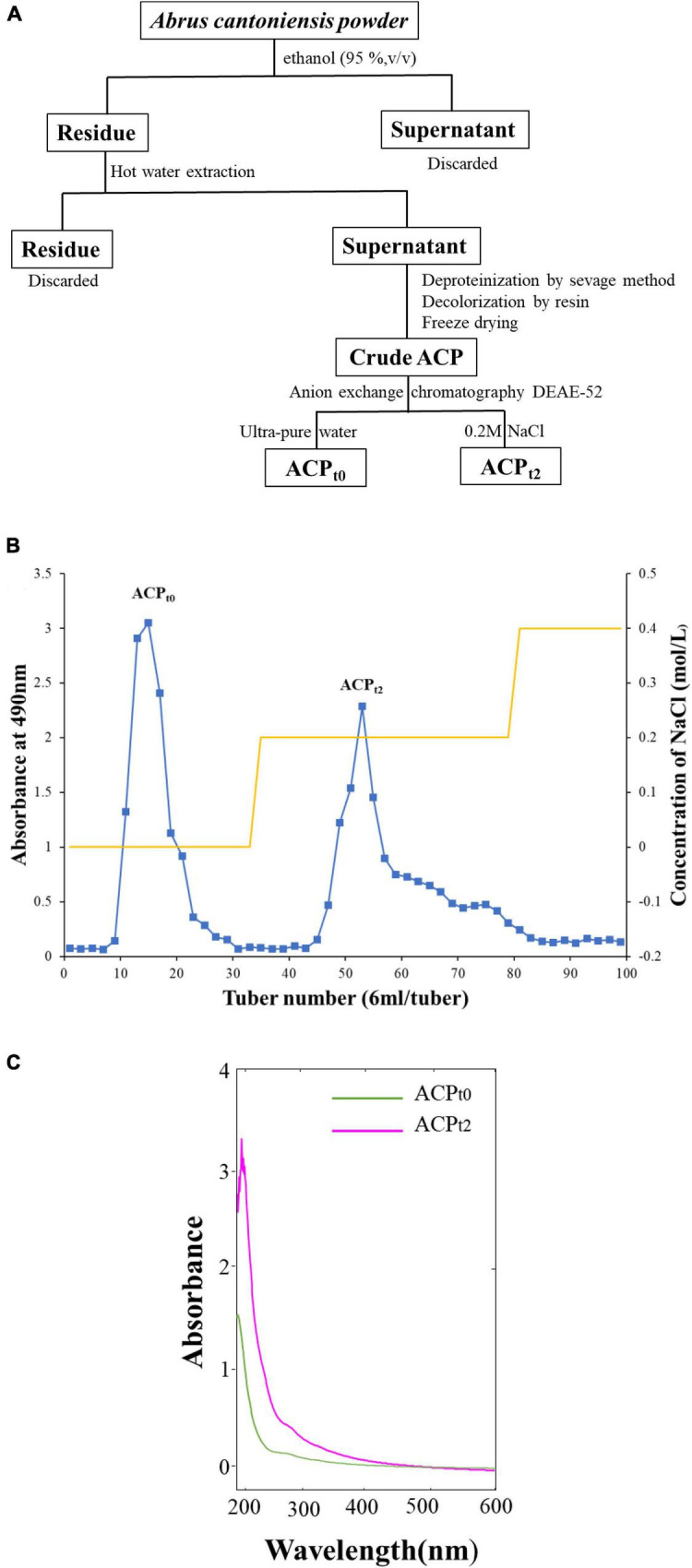
**(A)** The process of separation and purification of ACP. **(B)** Chromatography of crude ACP by DEAE-52 cellulose chromatography column. **(C)** UV spectrum of ACP_*t0*_ and ACP_*t2*_.

The water-ethanol extraction method was used to isolate the crude *Abrus cantoniensis* polysaccharides (21). To remove small molecules, the powder of *Abrus cantoniensis* was first processed with 95% ethanol, and then Savage deproteinization and resin decolorization. DEAE-52 anion exchange chromatography (2.6 × 50 cm) was used to further purify the crude polysaccharides, which was eluted with ultra-pure water and 0.2M NaCl into 2 peaks, the eluents were concentrated and dialyzed (cut-off Mw3500 Da) against distilled water for 36 h and vacuum-dried under freezing to obtain the refined ACP_*t*0_, ACP_*t*2_, respectively. The process of separation was shown in Figure 1A.

### Structural characterization of *Abrus cantoniensis* polysaccharides

#### Determination of contents of total sugar, protein and uronic acid

The total carbohydrate content was determined by the phenol-H_2_SO_4_ method (22). Using glucuronic acid as a standard, the total uronic acid content was determined by the carbazole sulfate method (23). The coomassie brilliant blue method, which uses bovine serum albumin as a reference, was used to determine the protein content. Visible absorbance was recorded with a UV–Vis spectrophotometer (Model SP-752, China).

#### Ultraviolet spectrum and Fourier transform infrared spectroscopy analysis

The purified ACP_*t*0_ and ACP_*t*2_ (2 mg each) were ground with KBr powder for FT-IR analysis and recorded between 4000 and 400 cm^–1^ on a Thermo Nicolet iS5 FT-IR (ThermoFisher, United States). The ultraviolet absorbance of the ACP_*t*0_ and ACP_*t*2_ aqueous solution (1 mg/mL) were scanned in the range of 200–600 nm by a U-6000PC spectrophotometer (Yuanxi, Shanghai, China).

#### Molecular weight and monosaccharide compositions analysis

The average molecular weight (MW) of ACP_*t*0_ and ACP_*t*2_ were analyzed by high-performance gel permeation chromatography (HPGPC) using an Agilent 1260 system (Agilent, United States) equipped with a refractive index detector (RID) and a TSK-GEL GMPWXL column (7.8 mm × 300 mm, 10μm) (Tosoh, Tokyo, Japan). For each run, polysaccharides samples (20 μL, 5.0 mg/mL) were injected at 40°C. Data were analyzed by GPC software and the molecular weight of ACP_*t*0_ and ACP_*t*2_ were determined using the standard curve obtained from dextran standards.

The monosaccharide compositions of ACP_*t*0_ and ACP_*t*2_ were analyzed by PMP pre-column derived method according to the previous report (24). In brief, the polysaccharides samples were hydrolyzed with 2 M trifluoroacetic acid (TFA) in an ampoule bottle at 120°C for 2 h. Then methanol was added into the hydrolyzate and dried with a nitrogen-blowing apparatus. The process was repeated three times to remove TFA. Next, the resulting residue was dissolved in distilled water and then the samples or monosaccharide standard mixture were mixed with 0.6 M NaOH and 0.5 M methanol solution of PMP. The reaction mixture was cooled to room temperature after incubating at 70°C for 1 h. added 0.3 M HCl to stop the derivative reaction and extracted by CHCl_3_ thrice. The PMP-labeled sugars were analyzed using an Agilent 1260 HPLC system with C18 column (4.6 mm × 250 mm, 5 μm Thermo, United States), and the chromatography conditions were as follows: temperature, 30°C; the mobile phase consisted of 87% 0.1 M phosphate solution (PH = 6.7) and 13% acetonitrile; detector, 245 nm. The monosaccharide compositions and content of ACP_*t*0_ and ACP_*t*2_ were identified according to the retention times and the calibration curve (peak area-concentration) of each monosaccharide standard, respectively.

#### Determination of a triple-helix structure

The spatial structure of ACP_*t*0_ and ACP_*t*2_ were analyzed by the Congo-red method with minor modifications (25). Polysaccharides samples (2 mg each) were dissolved in 1 mL of deionized water and mixed with 2 mL of Congo-red solution (100 μM). Then, 0.1 M phosphate buffer was dropped into the mixtures (PH = 8). The maximum absorption wavelength (λmax) of the complex were recorded using a UV-6000PC spectrophotometer (Yuanxi, Shanghai, China) in the wavelength range of 400–650 nm, which can determine the transition of maximum absorption wavelength of the mixtures.

#### Atomic force microscopy analysis

The polysaccharide samples (ACP_*t*0_ and ACP_*t*2_) were dissolved in ultrapure water (10 μg/mL). Sample solutions (5 μL each) were dropped onto clean mica sheets and dried at room temperature for 2 h. The surface molecular morphology of ACP_*t*0_ and ACP_*t*2_ were scanned by AFM (Bruker, United States).

### Determination of immunomodulatory activity

#### Isolation and culture of peritoneal macrophages

Specific-pathogen-free (SPF) mice (20 ± 2 g) were purchased from Changsha Tianqin Biotechnology Co., Ltd [certificate number: SCXK(Xiang)2019–0014, Hunan, China]. The entire experimental procedures were approved and supervised by the Animal Care and Welfare Committee of Guangxi University (Gxu-2021-116).

Peritoneal macrophages were extracted from mice’s abdominal cavity according to the reported methods (26). Briefly, the mice were intraperitoneally injected 1 mL of 0.5% starch broth medium. After two days, the mice were sacrificed and soaked in 75% alcohol for 5 min, then 6 mL of inactivated PBS was injected into the abdominal cavity and the abdomen was gently massaged for 2 min. Next, the peritoneal fluid was retracted, 2000 r/min, 5 min, then the cells were washed and stained with Trypan blue to confirm that the survival rate was above 95%. The concentration was adjusted to 1 × 10^6^ cells/mL with RPMI-1640 supplemented with 10% (v/v) fetal bovine serum (FBS). The cells were added to the cell culture plate and cultured in 37^°^C, 5% CO_2_ incubator.

#### Cytotoxicity of *Abrus cantoniensis* polysaccharides on macrophages

ACP_*t*0_ and ACP_*t*2_ were diluted to 11 concentrations in RMI1640 medium with a range of 1000∼0.98 μg/mL. 100 μL macrophages suspension were plated into 96-well plates with a density of 1 × 10^6^ cells per well, and cultured in 37^°^C, 5% CO_2_ incubator. After 4 h, macrophages were washed with PBS twice to remove the unadherent cells. Then 100 μL of various concentrations of samples were added, and cells cultured alone in RPMI1640 medium were used as control. After culturing for 44 h, 20 μL MTT solution (5 mg/mL) was added to each well for 4 h at 37^°^C. The supernatant was removed carefully and then 150 μL DMSO solution was added to each well. The plates were shaken for 10 min to completely dissolve the formazan crystals. The absorbance was recorded by a Microplate Reader at 570 nm (Thermo fisher, United States).

#### Measurement of reactive oxygen species generated by macrophages

The macrophages were plated in 96-well plates and cultured in 37^°^C, 5% CO_2_ incubator for 4 h. After removing the supernatant carefully, RMI1640 medium and various concentrations of ACP_*t*0_ and ACP_*t*2_ (3.91∼62.5 μg/mL) and LPS (10 μg/mL) were added to each well, respectively. After 44 h of incubation, all supernatants were carefully removed and 100 μL of DCFH-DA (10 μM) solution was added to each well, followed by incubation at 37°C for 20 min. After that, the supernatant was carefully removed and the cells were washed three times with PBS. The fluorescence intensity of each well was measured at 488 nm excitation and 525 nm emissions on a microplate reader.

#### Determination of the pinocytic activity of macrophages

The effect of ACP_*t*0_ and ACP_*t*2_ on the pinocytic activity of macrophages was determined by neutral red assay. The macrophages were seeded on 96-well plates and treated with different concentrations of ACP_*t*0_ and ACP_*t*2_, RMI1640 medium, and LPS, respectively. After 44 h of culture, the supernatant was removed. Each well was then filled with 100 μL of 0.1% neutral red solution, which was then incubated for 2 h at 37°C. Next, the supernatant was discarded and the cells in the 96-well plates were washed with PBS three times. Subsequently, cell lysate (100 μL per well) was added into each well for 2 h at room temperature. The optical density (OD) was recorded using a Microplate Reader at 540 nm.

#### Phagocytosis assay of fluorescein isothiocyanate-labeled ***Escherichia coli***

Vybrant phagocytosis assay kit was used to determine the phagocytic effect of two polysaccharide fractions on the phagocytosis fluorescein isothiocyanate (FITC)-labeled *Escherichia coli* at the concentration range of 3.91∼62.5 μg/mL. Macrophages were seeded on 24-well plates and treated with ACP_*t*0_, ACP_*t*2_, RMI1640 medium and LPS, respectively (described above). After that, 100 μL of the prepared fluorescent bioparticle suspension (1.0 mg/mL) was added to all the experimental wells and the plate was standed for 2 h at 37°C. Then the bioparticle loading suspension was removed from all of the wells carefully and the cells which phagocytize the fluorescent particles was washed three times with cold PBS. The trypan blue staining was to anchor the cells and then photographed under an inverted fluorescence microscope (EVOS M5000, Invitrogen, America).

#### Quantitative analysis of nitric oxide, iNOS and cytokines

The peritoneal macrophage was propagated in RMI1640 and stimulated with different concentrations of polysaccharide samples (3.91∼62.5 μg/mL) or LPS (10 μg/mL). 44 h of incubation later, the supernatants of the cells were obtained. Then, the levels of NO, iNOS, TNF-α, IL-6 and IL-1β were determined by the Griess reagent and ELISA kits, respectively.

#### RNA extraction and quantitative real-time polymerase chain reaction analysis

Macrophages were treated with different concentrations of polysaccharide samples or LPS for 24 h, respectively, according to the above method. The cells were collected for total RNA extraction using Trizol reagent. Then total RNA was reversed into cDNA by the StarScript II First-strand cDNA Synthesis Mix. Complementary DNA generated by reverse transcriptase encoding TNF-α, IL-1β and IL-6 genes was amplified by quantitative real-time polymerase chain reaction (qPCR). The β-actin was utilized as the internal reference to normalize the gene expression. The 2^–Δ^ ^Δ^ *^Ct^* method was used to calculate the expression levels of mRNA. The blank control was treated with RMI1640 medium alone and the positive control group was treated with LPS (10 μg/mL). Gene amplification was performed by Real Star Green Fast Mixture on the Light Cycler 96 System (Roche). The primer sequences used in this study (Sangon Biotech Co., Ltd, China) are shown in Table 1.

**TABLE 1:** Sequence of primers.

Genes		Primer sequence (5′ to 3′)
β-actin	Forward	GAGGGAAATCGTGCGTGAC
	Reverse	GCTGGAAGGTGGACAGTGAG
TNF-α	Forward	CTCATTCCTGCTTGTGGC
	Reverse	CACTTGGTGGTTTGCTACG
IL-6	Forward	TTCCATCCAGTTGCCTTC
	Reverse	GTAATTAAGCCTCCGACT
IL-1β	Forward	GTTCCCATTAGACAACTGC
	Reverse	AGATTCTTTCCTTTGAGGC
TLR2	Forward	ACCCGCCCTTTAAGCTGTGT
	Reverse	TCGTACTTGCACCACTCGCT
TLR4	Forward	TCTGGGGAGGCACATCTTCT
	Reverse	AGGTCCAAGTTGCCGTTTCT
MyD88	Forward	TCATGTTCTCCATACCCTTGGT
	Reverse	AAACTGCGAGTGGGGTCAG
MAPK	Forward	TGACCCTTATGACCAGTCCTTT
	Reverse	GTCAGGCTCTTCCACTCATCTAT
AKT	Forward	TGAAGCTACTGGGCAAGGG
	Reverse	AAAGCAGAGGCGGTCGTG

## Result and discussion

### Extraction and purification of polysaccharides

As shown in Figure 1B, the crude ACP was further purified by DEAE-52 Cellulose anion-exchange chromatography, and two mainly independent elution peaks were obtained. The content of carbohydrate of ACP_*t*0_ and ACP_*t*2_ were 99.6% and 98.8%, respectively. The UV spectrum of two polysaccharides showed no absorption peaks at 260 and 280 nm, indicating that the absence of protein and nucleic acid in ACP_*t*0_ and ACP_*t*2_ (Figure 1C). The uronic acid contents of ACP_*t*0_ and ACP_*t*2_ were 15.9% and 27.9% (Table 2). From the subsequent FT-IR analysis and monosaccharide composition analysis, the uronic acid can also be obviously detected, but the proportion is not consistent. Monosaccharide composition tests showed that ACP_*t*0_ contained a large amount of glucose. Therefore, it is speculated that the high content of uronic acid may be caused by the oxidation of glucose in the polysaccharide to glucuronic acid in ACP_*t*0_ using sulfuric acid-carbazole method.

**TABLE 2:** The total sugar, uronic acid, and protein contents, and monosaccharide compositions molecular weight of the ACP_*t0*_ and ACP_*t2*_.

Fraction	Total carbohydrate (%)	Uronic acid (%)	Protein (%)	MW (kDa)	Monosaccharide composition (%)							
					Man (%)	Rha (%)	GlcUA (%)	GalUA (%)	Glc (%)	Gal (%)	Xyl (%)	Ara (%)
ACP_*t0*_	98.8	15.9	0.09	26.0	1.4	–	1.6	1.6	83.1	6.1	–	1.6
ACP_*t2*_	99.6	27.9	0.11	145.6 /8.9	8.8	1.7	3.9	22.2	11.0	25.6	6.3	16.6

– Not detected.

### Structural characterization of *Abrus cantoniensis* polysaccharides

#### Molecular weight and monosaccharide composition

**FIGURE 2:**
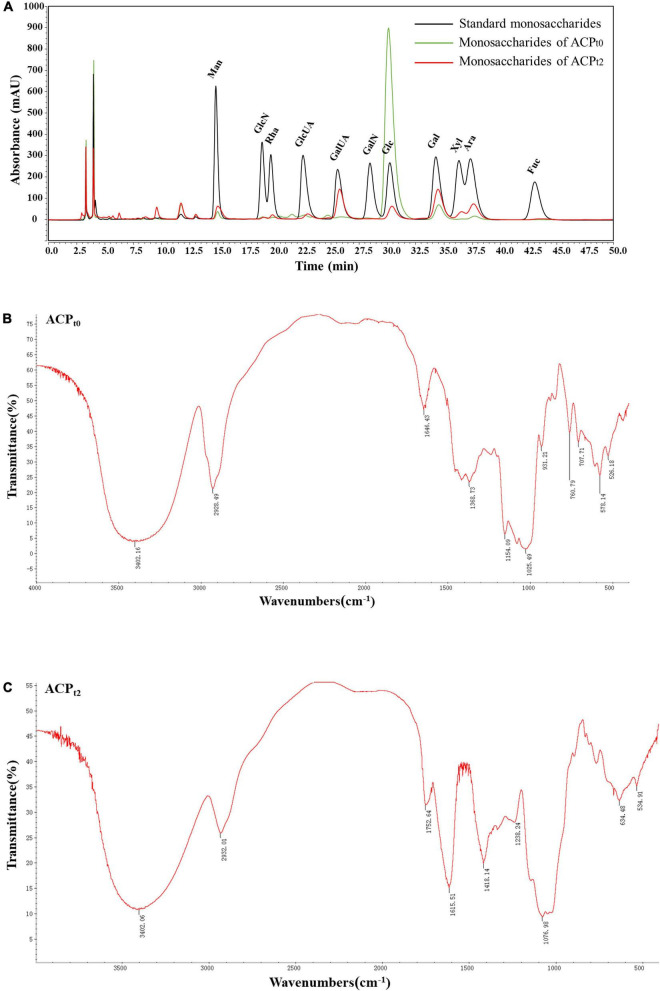
**(A)** Ion chromatogram data of monosaccharide composition of ACP_*t0*_ and ACP_*t2*_, Fourier-transform infrared (FT-IR) spectra of ACP_*t0*_ **(B)**, and ACP_*t2*_ **(C)** in the range of 4000–400 cm^–1^.

ACP_*t*0_ exhibited one main elution peak with an estimated molecular weight of 26.0 kDa. While, the molecular weight of ACP_*t*2_ with two peaks was calculated at 145.6 kDa and 8.9 kDa. The monosaccharide composition of ACP_*t*0_ and ACP_*t*2_ were analyzed by PMP pre-column derived method. As shown in Figure 2A, results indicated ACP_*t*0_ was mainly composed of glucose (83.1%), galactose (6.1%), and a small amount of galactose uronic acid, glucuronic acid, arabinose and mannose, signifying that glucose was probably the backbone of ACP_*t*0_. In contrast, ACP_*t*2_ contains mainly galactose (25.6%), galactose uronic acid (22.2%), arabinose (16.6%), glucose (11.0%), and a small amount of mannose, xylose, glucuronic acid, rhamnose (Table 2).

#### Infrared spectroscopic Fourier transform infrared spectroscopy analysis

Due to each group’s distinctive absorption, IR spectrum analysis is a useful tool for understanding polysaccharide structure. FT-IR spectra of ACP_*t*0_ and ACP_*t*2_ were shown in Figures 2B,C, respectively. The results showed the broad peak at 3402.16 cm^–1^ for ACP_*t*0_ and 3402.06 cm^–1^ for ACP_*t*2_ corresponded to the -OH stretching vibration, and 2928.49 cm^–1^ for ACP_*t*0_ and 2932.01 cm^–1^ for ACP_*t*2_ attributed to the stretching vibration of -C-H, which indicated the presence of intermolecular hydrogen bonds in the molecular structure (27). 1646.43 cm^–1^ and 1368.73 cm^–1^ for ACP_*t*0_ and 1615.51 cm^–1^ and 1418.14 cm^–1^ for ACP_*t*2_ probably caused by carbonyl or aldehyde or carboxyl (28, 29). Assigned to the C = O stretching vibration of acetyl or carboxylic acid ester, the peaks at 1752.64 cm^–1^ in ACP_*t*2_ indicated that some carboxyl groups were esterified (30). The O = S = O deformation vibration is indicated by the band at 1238.24 cm^–1^, which may be an indication of the presence of sulfates in the ACP_*t*2_ (31). The absorption peaks at 1025.49 cm^–1^ and 1154.09 cm^–1^ for ACP_*t*0_, 1076.98 cm^–1^ for ACP_*t*2_ are supposed to the characteristic peaks of pyran-glycosides (32). The IR spectrum of ACP_*t*0_ at 1500–500 cm^–1^ was different from those of ACP_*t*2_, which may be attributed to the difference in their monosaccharide compositions.

#### Chain conformation

**FIGURE 3:**
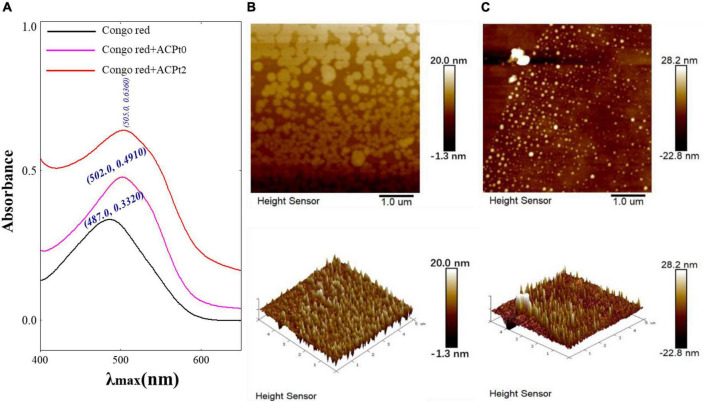
**(A)** Maximum absorption wavelength of Congo red + ACP_*t0*_ or ACP_*t2*_ complex and Congo red; AFM spectra of ACP_*t0*_ **(B)** and ACP_*t2*_ **(C)**, planar image (upper panels, scan size: 5 μm), cubic image (lower panel, scan size: 5 μm).

Congo red is an acidic dye, which can form a complex with triple-helix polysaccharides. In a suitable alkaline condition, the λmax of the complex is red-shifted compared with Congo red. Therefore, Congo red experiment can corroborate the existence of triple helix structure in polysaccharides (33). As shown in Figure 3A, the bathochromic shift of λmax value of ACP_*t*0_ or ACP_*t*2_-Congo red complex are 15 nm and 18 nm, respectively compared with Congo red. The results indicated that ACP_*t*0_ and ACP_*t*2_ could form a complex with Congo red, indicating the existence of triple helix conformation in ACP_*t*0_ and ACP_*t*2_.

#### Spectroscopy molecular surface morphology

The AFM is a powerful technique of structure characterization analysis, which can be used to observe the morphology and size of macromolecular chains in samples under room temperature and pressure. AFM has gradually become a vital approach to explore the structures of biological macromolecules, such as polysaccharides and DNA (34). As is shown in Figures 3B,C, spherical shell morphology was clearly observed (upper panels). The diameters of ACP_*t*0_ and ACP_*t*2_ were estimated to be 11.20–48.60 nm, 60.30–280 nm, respectively, which indicated that ACP_*t*0_ and ACP_*t*2_ have the potential of application in the drug carrier field. ACP_*t*0_ aggregated into dispersed spherical particles significantly smaller than ACP_*t*2_ in aqueous solution. The 3D figures revealed the island structure of two polysaccharides. Many island-like protrusions with heights between -22.80 and 28.20 nm were discovered on the surface of ACP_*t*0_ and ACP_*t*2_. The surface height of ACP_*t*0_ is slightly greater than ACP_*t*2_. It indicated that the biological activity of polysaccharides was related to the diameter and surface height of spherical particles focused in aqueous solution.

### Determination of immunomodulatory activity

#### Cytotoxicity of *Abrus cantoniensis* polysaccharides on macrophages

**FIGURE 4:**
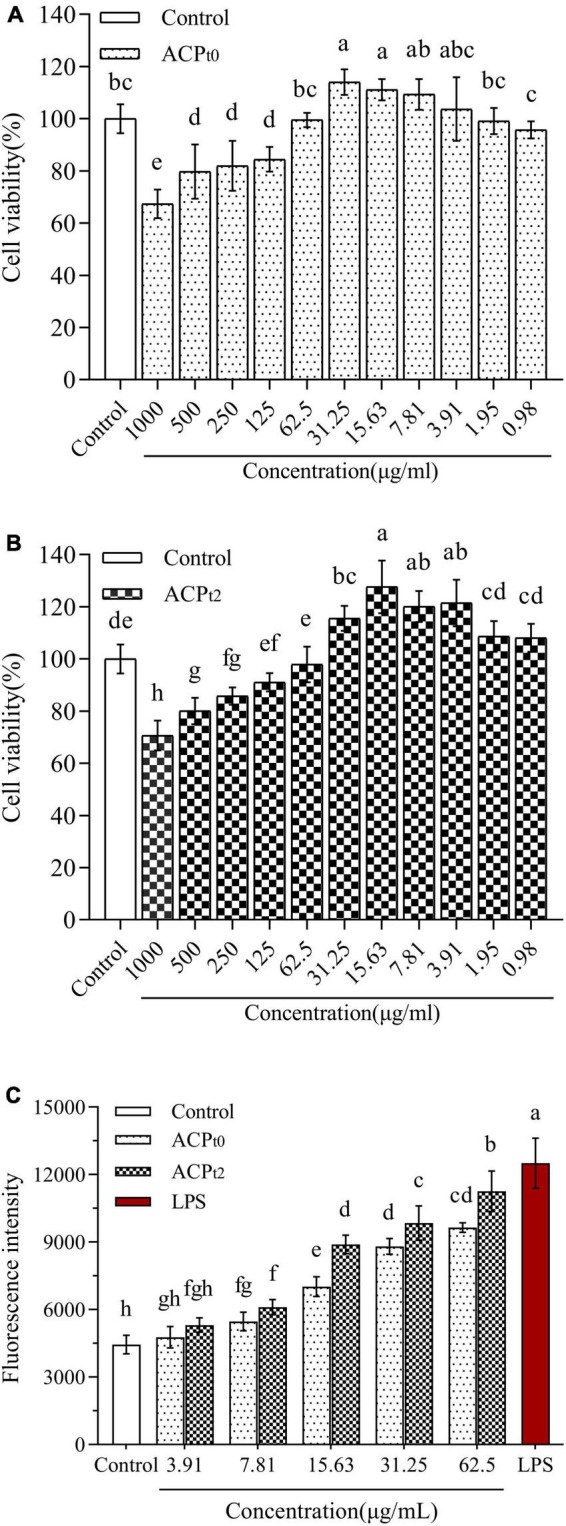
The effects of ACP_*t0*_ **(A)** and ACP_*t2*_ **(B)** treatment on the viability of macrophages. Effect of different concentrations of ACP_*t0*_ and ACP_*t2*_ treatment on ROS generation of macrophages **(C)**. The different letters in the column indicate statistically significant differences (*P* < 0.05).

The effects of ACP_*t*0_ and ACP_*t*2_ on the proliferation of peritoneal macrophages were investigated at concentrations of 1000∼0.98 μg/mL. As shown in Figure 4, ACP_*t*0_ and ACP_*t*2_ within a given concentration range had no inhibitory effects on the proliferation of macrophages. Compared with normal control, the growth of macrophages was inhibited notably when the concentration of ACP_*t*0_ was greater than 62.5 μg/mL (*P* < 0.05), and when concentration exceeded 125 μg/mL, ACP_*t*2_ could significantly suppress the proliferation of macrophages (*P* < 0.05). In the subsequent experiments, the concentration range of the two polysaccharides was set as 3.91∼62.5 μg/mL to facilitate comparison at the same level.

#### Measurement of reactive oxygen species generated by macrophages

In this study, fluorescence probe DCFH-DA method was used to measure the changes of ROS secretion of macrophages after treatment with different concentrations of ACP_*t*0_ and ACP_*t*2_ (3.91∼62.5 μg/mL) for 24 h. These probes can easily infiltrate cell membranes and enter cells, and then be hydrolyzed by intracellular esterase to produce 2’,7’-dichlorodihydrofluorescein (DCFH) without fluorescence. The DCFH was subsequently oxidized by intracellular ROS to 2’,7’-dichlorodihydrofluorescein (DCF) with green fluorescence (35). Therefore, fluorescence intensity is positively correlated with intracellular ROS levels. As shown in Figure 4C, the mean fluorescence intensity (ROS levels) of blank control group was 4446.75 ± 415.06, while treatment with LPS (10 μg/mL) and two samples increased ROS production. However, low concentration of ACP_*t*0_ and ACP_*t*2_ (3.91 μg/mL) did not significantly increase ROS secretion (*P* > 0.05). With the increase in the concentrations of ACP_*t*0_ and ACP_*t*2_, ROS levels increases in a dose-dependent manner. When the concentration of ACP_*t*0_ and ACP_*t*2_ reached 62.5 μg/mL, the ROS secretion levels were 9642.25 ± 209.48 and 11257.50 ± 896.39, respectively, increased by 116.84% and 153.16% compared with blank control group (*P* < 0.01), still significantly lower than that of LPS group (181.23%) (*P* < 0.05). These results suggest that treatment with ACP_*t*0_ and ACP_*t*2_ could modulate the immune activity of macrophages moderately.

#### Effects of *Abrus cantoniensis* polysaccharides on the pinocytic and phagocytic activities of macrophages

**FIGURE 5:**
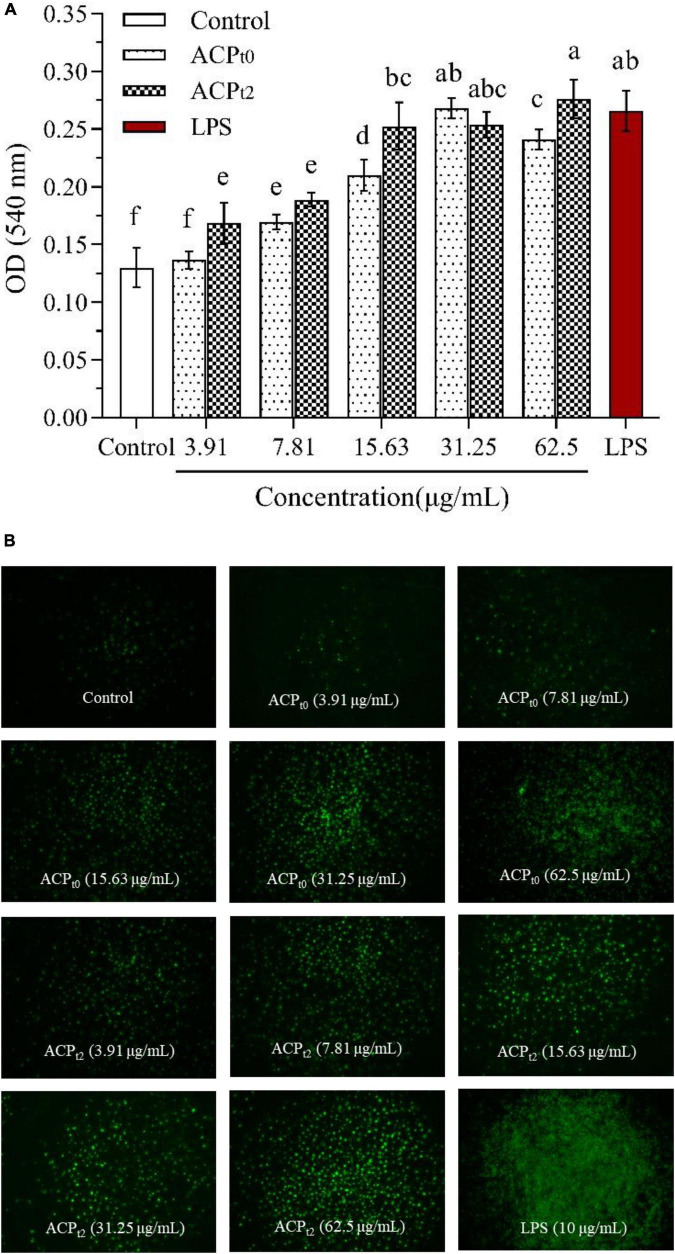
Pinocytiosis rate of ACP_*t0*_ and ACP_*t2*_ on taking neutral red of macrophages **(A)**. Fluorescence microscopic images of macrophages phagocytosing FITC-labeled *E. coli* **(B)**. The different letters in the column indicate statistically significant differences (*P* < 0.05).

As shown in Figure 5, except for ACP_*t*0_ at 3.91 μg/mL, the phagocytosis of neutral red by macrophages was significantly enhanced after 48 h stimulation with different concentrations of ACP_*t*0_ and ACP_*t*2_ (3.91∼62.5 μg/mL) (*P* < 0.05). The absorbance value (OD_540_) of cells pretreated by ACP_*t*0_ and ACP_*t*2_ reached the maximum (OD_540_ = 0.268, 0.276) at 31.25, 62.5 μg/mL, which was 106.2%, 112.3% greater (*P* < 0.05) than that of blank control group (OD_540_ = 0.130), respectively. The absorbance value (OD_540_) of cells treated by LPS reached 0.266, which was significantly higher than that in the normal control (*P* < 0.05). The results showed that ACP_*t*0_ and ACP_*t*2_ could promote the uptake of neutral red, and significantly enhance the phagocytosis of macrophages. Especially, the induction effect of ACP_*t*2_ on macrophages was stronger than ACP_*t*0_.

Additionally, the effects of ACP_*t*0_ and ACP_*t*2_ on the phagocytic uptake of *E. coli* (FITC-labeled) in macrophages were visualized under inverted fluorescence microscope. The image of the control group showed the darkest fluorescence, indicating little phagocytosis of *E. coli* by the untreated cells. When the concentrations of ACP_*t*0_ and ACP_*t*2_ increased, the clear increase in fluorescence was observed. The fluorescence intensity of ACP_*t*2_-treated cells was higher than that of ACP_*t*0_ at the same concentration.

While a significant difference was observed in the brightness and density of macrophages treated with LPS, which was markedly greater than that in the ACP_*t*0_ and ACP_*t*2_ treatment groups. These results indicate that ACP_*t*0_ and ACP_*t*2_ can regulate immune activity and do not over-activate macrophages. The effect of ACP_*t*2_ is superior to ACP_*t*0_, which is consistent with the result of neutral red experiment.

#### Quantitative analysis of nitric oxide, iNOS

**FIGURE 6:**
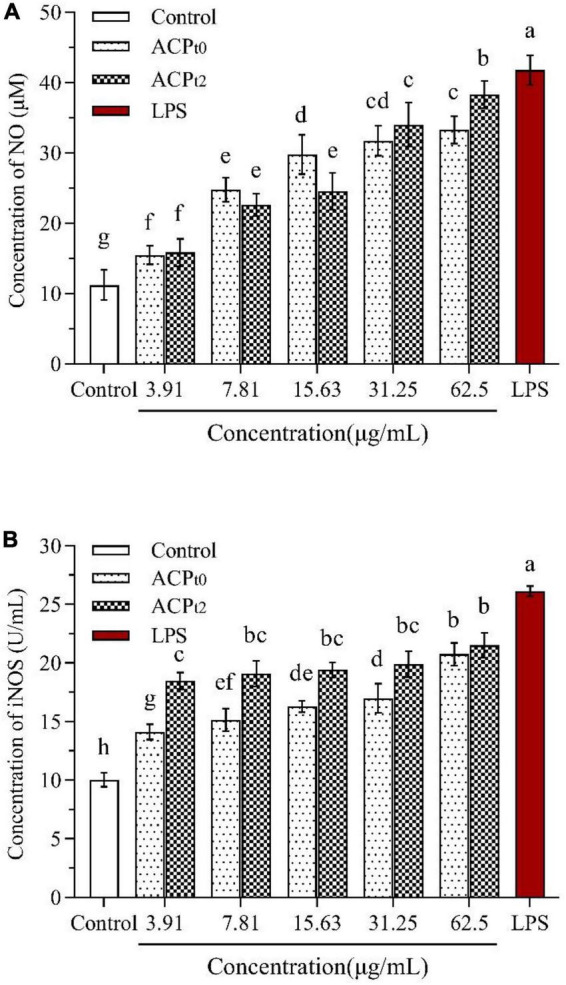
Effects of ACP_*t0*_ and ACP_*t2*_ on NO secretion **(A)**, the activity of iNOS **(B)** in macrophages. The different letters in the column indicate statistically significant differences (*P* < 0.05).

Compared with the blank control, NO levels were significantly higher (*P* < 0.05) in concentration-dependent after activation of macrophages by different doses of ACP_*t*0_ and ACP_*t*2_ (3.91∼62.5 μg/mL) (Figure 6A). When the concentration of polysaccharides increased from 3.91 to 62.5 μg/mL, the level of NO induced by ACP_*t*0_ gradually increased from 15.46 ± 1.32 to 33.26 ± 1.93 μmol/L. While the levels of NO induced by ACP_*t*2_ gradually increased from 15.85 ± 1.93 μmol/L to 38.29 ± 1.93 μmol/L. The results revealed ACP_*t*2_ on NO production from macrophages was stronger than that of ACP_*t*0_ under high concentrations. However, the levels of NO at all concentrations of ACP_*t*0_ and ACP_*t*2_ were markedly lower than those stimulated by LPS (*P* < 0.05).

As shown in Figure 6B, the activity of iNOS was 10.04 ± 0.59 U/mL in the blank control, and the activity of iNOS induced by LPS reached 26.12 ± 0.44 U/mL, which was significantly higher than that in the blank control (*P* < 0.05). ACP_*t*0_ and ACP_*t*2_ at all concentrations significantly enhanced the activity of iNOS in macrophages compared with the blank control (*P* < 0.05). The activity of iNOS induced by ACP_*t*0_ increased in a dose-dependent manner, and reached the highest levels of 17.22 ± 0.68 U/mL at 62.5 μg/mL. Whereas there was no significant difference in the activity of iNOS induced by ACP_*t*2_ at each concentration. However, it was worth noting that the activity of iNOS induced by ACP_*t*2_ at the concentrations of 3.91, 7.81, 15.63, 31.25 μg/mL was stronger than the activity of iNOS stimulated by ACP_*t*0_ (*P* < 0.05).

#### Quantitative analysis of cytokines and their mRNA expression in macrophages

**FIGURE 7:**
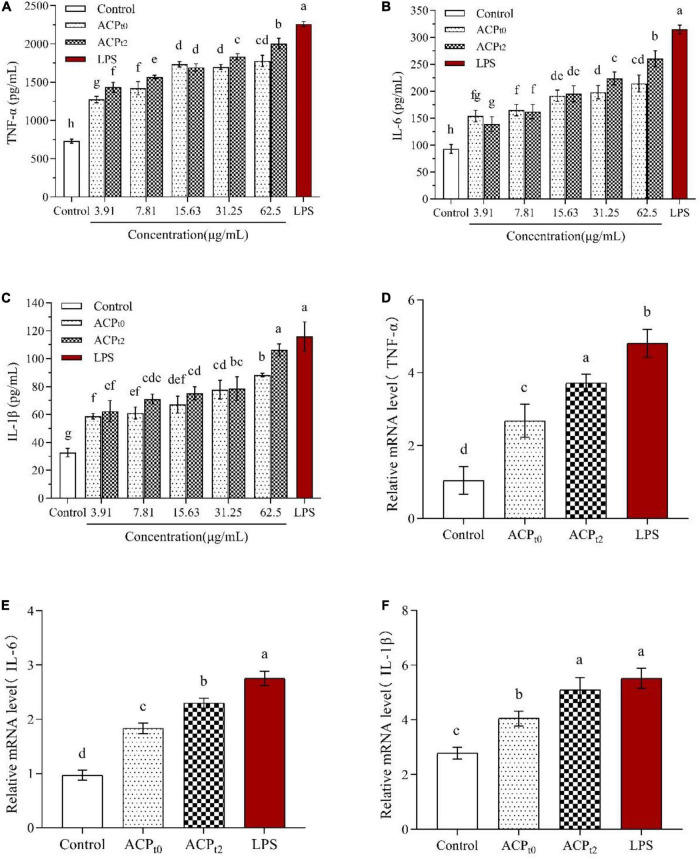
Effects of ACP_*t0*_ and ACP_*t2*_ on secretion of TNF-α **(A)**, IL-6 **(B)**, and IL-1β **(C)**, relative mRNA expression levels of TNF-α **(D)**, IL-6 **(E)**, and IL-1β **(F)** in macrophages. The different letters in the column indicate statistically significant differences (*P* < 0.05).

The effects of ACP_*t*0_ and ACP_*t*2_ on the levels of TNF-α, IL-6 and IL-1β in macrophages were detected by ELISA. As shown in Figure 7, ACP_*t*0_ and ACP_*t*2_ at concentrations of 3.91∼62.5 μg/mL markedly promoted the secretion of TNF-α, IL-6 and IL-1β in macrophages compared with the normal control (*P* < 0.05). The secretion of TNF-α and IL-6 in macrophages stimulated by ACP_*t*2_ increased with significant dosage effects, while there was no obvious difference in the ACP_*t*0_ group at the concentrations ranging from 15.63 to 62.5 μg/mL. With the increase of samples concentrations, IL-1β secretions stimulated by ACP_*t*0_ and ACP_*t*2_ gradually increased and reached the highest level, and at 62.5 μg/mL, and ACP_*t*0_ and ACP_*t*2_ were 88.302 ± 1.34 pg/mL and 106.267 ± 4.38 pg/mL, respectively. However, except for the effect of ACP_*t*2_ on IL-1β secretion by macrophages at 62.5 μg/mL, the productions of TNF-α, IL-6 and IL-1β from macrophages activated by ACP were lower than in the LPS-treated group (*P* < 0.05). Notably, overproduction and excessive cytokines are considered to induce inflammation, which is harmful to the organisms. Results indicated that both ACP_*t*0_ and ACP_*t*2_ can activate the immune system by stimulating macrophages to secrete TNF-α, IL-6 and IL-1β.

To further verify whether ACP_*t*0_ and ACP_*t*2_ modulate the release of cytokines by upregulating gene expression, the mRNA expression levels of TNF-α, IL-6 and IL-1β in macrophages were detected by qRT-PCR and the results were calculated using 2^–ΔΔ*Ct*^ method. Since the results showed a better treatment effect at 62.5 μg/mL, the concentration of the samples was selected 62.5 μg/mL for further study. The results are shown in Figures 7D–F, compared to the blank control group, ACP_*t*0_ and ACP_*t*2_ distinctly enhanced the expression levels of TNF-α, IL-6 and IL-1β at 62.5 μg/mL (*P* < 0.05). Except for the mRNA level of IL-1β in the ACP_*t*2_ group, the LPS group was significantly higher than that in ACP_*t*0_ and ACP_*t*2_ groups (*P* < 0.05). The results indicated that cytokine production was positively correlated with the expression of related genes, and ACP_*t*0_ and ACP_*t*2_ could upregulate cytokine secretion and related mRNA expression in macrophages.

#### *Abrus cantoniensis* polysaccharides activated signaling pathway of macrophages

**FIGURE 8:**
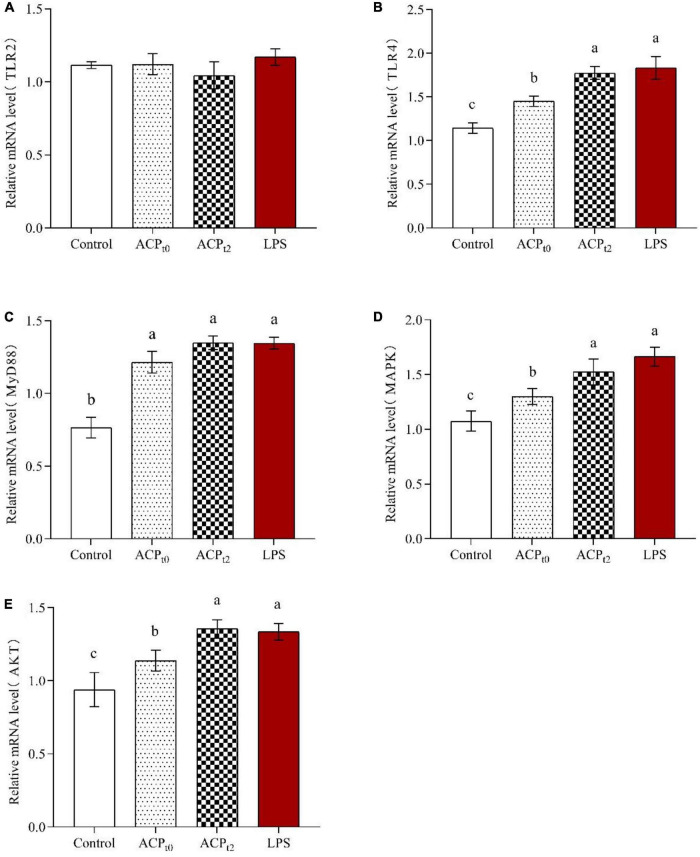
Effects of ACP_*t0*_ and ACP_*t2*_ on relative mRNA expression levels of TLR2 **(A)**, TLR4 **(B)**, MyD88 **(C)**, MAPK **(D)**, and AKT **(E)** in macrophages. The different letters in the column indicate statistically significant differences (*P* < 0.05).

The expression of TLR2, TLR4, MyD88, MAPK, and AKT in macrophages was detected by qRT-PCR to determine the regulatory role of ACP_*t*0_ and ACP_*t*2_ on macrophage-related genes. As shown in Figure 8, ACP_*t*0_, ACP_*t*2_ and LPS groups remarkably upregulated the levels of TLR4 compared to the blank control group (*P* < 0.05), while there was no effect on the expression of TLR2, suggesting that TLR4 were the major pattern recognition receptors of ACP in macrophages. Meanwhile, the stimulation of LPS, ACP_*t*0_ and ACP_*t*2_ resulted in a significant increase in the expression levels of MyD88, MAPK and AKT (*P* < 0.05). These results showed that ACP_*t*0_ and ACP_*t*2_ can activate macrophages by recognizing TLR4 to regulate key genes on the MyD88/Akt/MAPKs pathway, thereby enhancing the immune response of mouse macrophages.

## Discussion

In this study, the physicochemical properties and immunomodulatory activities of two novel polysaccharides (ACP_*t*0_ and ACP_*t*2_) from *Abrus cantoniensis* were determined. The results showed that ACP_*t*0_ and ACP_*t*2_ possessed excellent immunomodulatory activities.

Reactive oxygen species (ROS) are directly or indirectly transformed by oxygen radicals and their derivatives. As the second messenger in many signaling pathways, ROS, especially endogenous ROS, could regulate major molecular signaling pathways, such as MAPK pathway and mitochondria-mediated casepase apoptosis pathway, thus affecting the body’s differentiation, metabolism, cell proliferation, cell death and other important cellular activities (36). When inflammation occurs, macrophages and neutrophils are immediately activated, leading to the strengthening of respiration in the body, increasing oxygen consumption and producing ROS, which can participate in the synthesis of a series of inflammatory factors and enhance the phagocytosis ability of macrophages to kill bacteria and other foreign substances (37). Therefore, ROS can be used as a valid biomarker to reflect the immunomodulatory effect of ACP on macrophages. In this study, different concentrations of ACP_*t*0_ and ACP_*t*2_ can significantly increase the level of ROS in macrophages, which indicated that ACP could modestly modulated the immune activity of macrophages. Huo reported that polysaccharides (HSP-3) extracted from *Huangshui* can also remarkably stimulate ROS secretion in THP-1 cells (38).

Pinocytic and phagocytic capacities, as the fundamental defense mechanisms of macrophages, act an extremely key role in the defense response to pathogens and in maintaining homeostasis in the body. Therefore, pinocytic and phagocytic capacities can directly reflect the activity of macrophages and is a significant index to evaluate the immune function of the body (39). To investigate the effects of ACP_*t*0_ and ACP_*t*2_ on macrophages, pinocytic activity was quantitatively determined by the uptake of neutral red test, and the phagocytosis was visualized by FITC-labeled *E. coli* in fluorescence microscopy. In the present study we found that ACP_*t*0_ and ACP_*t*2_ could enhance the pinocytic and phagocytic capacities of macrophages. Besides, other plant polysaccharides, such as *Pueraria lobata Ohwi* polysaccharides (Ge-1) (12) and *Paeonia suffruticosa Andr* polysaccharides (PSAP) (40), can also significantly promote the phagocytosis of neutral red and FITC-labeled *E. coli* by macrophages.

Activated macrophages are able to secrete a range of chemokines and cytokines. NO as a signaling molecule of physiological changes, plays a vital role in cytotoxicity, immune regulation and intracellular bactericidal function of macrophages (41). iNOS can catalyze the conversion of L-arginine to NO in activated macrophages. Many studies show that plant polysaccharides can activate macrophages and enhance the NO level and iNOS activity of macrophages. The polysaccharide isolated from *Huangshui* could increase the level of NO and an acidic polysaccharide from *Citrus grandis* could promote iNOS expression in macrophages (38, 42). In this study, after treatment with ACP_*t*0_ and ACP_*t*2_, the NO secretion and iNOS activity of macrophages enhance markedly.

Cytokines are produced by immune cells stimulated by antigens or mitogens (43). TNF-α, as an immunomodulatory factor, can not only activate immune regulation and mediate inflammatory reactions, but also induce expressions of chemokines on macrophages and neutrophils, enhance cell trafficking. Moreover, TNF-α can enhance phagocytic effects of macrophages to kill exogenous pathogens or inhibit tumor cells (44). IL-6 is a pleiotropic cytokine, which can transmit messages between immune cells and participate in the body’s immune defense. IL-1β is involved in a variety of autoimmune inflammatory and immune responses and can stimulate the secretion of IL-6 (45). The rise expression of TNF-α, IL-6 and IL-1β in ACP_*t*0_ and ACP_*t*2_ groups was observed in our study, which means the macrophages got activated. Meanwhile, there was a similar trend in the mRNA expression of cytokines. The results indicated that ACP_*t*0_ and ACP_*t*2_ can promote the secretion of TNF-α, IL-6 and IL-1β by regulating the mRNA expression of cytokines.

Polysaccharides as macromolecules cannot cross the cell membrane directly. Studies have shown that polysaccharides can first recognize Toll-like receptors (TLRs) on the surface of macrophages, activating various intracellular signaling pathways, causing a series of signal cascade reactions and regulating the expression of related genes (46, 47). Previous studies have shown that TLR4, a classical endotoxin receptor, recognizes a variety of natural polysaccharides (48–50). Pu et al. found that *Solanum nigrum Linne* polysaccharide (SNLP) upregulated gene expression at important nodes in the TLR4-MyD88 signaling pathway, leading to changes of cytokines (TNF-α and IL-6), exerting immune effect (51). There are key genes in the intracellular signaling pathway, such as MyD88, Akt and MAPKs. MyD88 is an important bridging protein in the signaling pathway of TLRs and mainly mediates the expression and secretion of inflammatory cytokines in immune cells. The Akt signaling pathway can be involved in regulating cell proliferation and apoptosis processes. In addition, MAPKs signaling pathway is able to phosphorylate related cytoplasmic proteins, which can activate a variety of related transcription factors in the nucleus and phosphorylate them to promote cell proliferation (52). The expression of TLR4, MyD88, MAPK and AKT of macrophages in ACP_*t*0_ and ACP_*t*2_ groups increased, suggesting that ACP can activate macrophages by recognizing TLR4 on the macrophage surface, which in turn regulates key genes in the MyD88/Akt/MAPKs signaling pathway, thereby enhancing the immune response of mouse macrophages.

## Conclusion

Two polysaccharide fractions (ACP_*t*0_ and ACP_*t*2_) with average molecular weight of 26.0 kDa and 145.6/8.9 kDa were purified from *Abrus cantoniensis*. Then the physicochemical properties and immunomodulatory activities of ACP_*t*0_ and ACP_*t*2_ were investigated and compared. ACP_*t*0_ and ACP_*t*2_ possessed significant immunomodulatory activity *in vitro* in terms of elevating the proliferation, enhancing the pinocytic and phagocytic capacity and increasing the secretion of ROS, NO and iNOS in macrophage. Meanwhile, two polysaccharides promoted the secretion of cytokines (TNF-α, IL-6 and IL-1β) by activating the corresponding mRNA expression in macrophage. Besides, TLR4 was identified as the main membrane receptors, and the immunomodulatory activity of ACP on macrophage was mainly through MyD88/AKT/MAPKs signaling pathways. In addition, immunomodulatory activities of ACP_*t*2_ were stronger than that of ACP_*t*0_. These findings suggested ACP_*t*0_ and ACP_*t*2_ can be a potential immunostimulator for application in functional foods and pharmaceutical fields.

## Data availability statement

The raw data supporting the conclusions of this article will be made available by the authors, without undue reservation.

## Author contributions

HS initiated the project and supervised and conducted the experimental work. DQ and WS were responsible for designing experimental ideas, data analysis, protocol designing, and draft editing. SL and HH conducted the experimental work and processed the data. All authors contributed to the article and approved the submitted version.
